# Identification of molecular mechanisms for cellular drug resistance by combining drug activity and gene expression profiles

**DOI:** 10.1038/sj.bjc.6602699

**Published:** 2005-07-12

**Authors:** L Rickardson, M Fryknäs, S Dhar, H Lövborg, J Gullbo, M Rydåker, P Nygren, M G Gustafsson, R Larsson, A Isaksson

**Affiliations:** 1 Department of Medical Sciences, Division of Clinical Pharmacology, Uppsala University Hospital, S-751 85 Uppsala, Sweden; 2 Department of Genetics and Pathology, Uppsala University, S-751 85 Uppsala, Sweden; 3 Department of Oncology, Radiology and Clinical Immunology, Uppsala University, S-751 85 Uppsala, Sweden; 4 Department of Engineering Sciences, Uppsala University, S-751 85 Uppsala, Sweden

**Keywords:** chemotherapy, drug resistance, gene microarray

## Abstract

Acquired drug resistance is a major problem in cancer treatment. To explore the genes involved in chemosensitivity and resistance, 10 human tumour cell lines, including parental cells and resistant subtypes selected for resistance against doxorubicin, melphalan, teniposide and vincristine, were profiled for mRNA expression of 7400 genes using cDNA microarray technology. The drug activity of 66 cancer agents was evaluated on the cell lines, and correlations between drug activity and gene expression were calculated and ranked. Hierarchical clustering of drugs based on their drug–gene correlations yielded clusters of drugs with similar mechanism of action. Genes correlated with drug sensitivity and resistance were imported into the PathwayAssist software to identify putative molecular pathways involved. A substantial number of both proapoptotic and antiapoptotic genes such as signal transducer and activator of transcription 1, mitogen-activated protein kinase 1 and focal adhesion kinase were found to be associated to drug resistance, whereas genes linked to cell cycle control and proliferation, such as cell division cycle 25A and signal transducer of activator of transcription 5A, were associated to general drug sensitivity. The results indicate that combined information from drug activity and gene expression in a resistance-based cell line panel may provide new knowledge of the genes involved in anticancer drug resistance and become a useful tool in drug development.

Chemotherapy is an important modality for the treatment of malignant tumours. However, for the majority of cancer patients, treatment with established anticancer drugs produces dissatisfactory long-term effects and drug activity is highly variable both between and within different diagnoses (Nygren, 2001). Genes affecting chemosensitivity are involved in drug transport, drug metabolism, DNA synthesis and repair, cell survival and apoptosis (Marie, 2001; Pommier *et al*, 2004). Since many different signalling pathways are involved, there is an urgent need for high-efficacy drugs with novel mechanisms of action targeting the key genes.

Rapid cell-based methods for high-throughput and focused screening based on drug-response analysis in a panel of cell lines have proven to be important tools in anticancer drug discovery and early evaluation (Paull *et al*, 1989; Boyd and Paull, 1995; Dhar *et al*, 1996; Weinstein *et al*, 1997). Research performed at the National Cancer Institute (NCI) has shown that a drug activity profile acquired from a nonclonogenic growth inhibition assay on a panel of 60 parental human cancer cell lines can provide important information on the mechanism of action of various compounds. Robust and accurate mechanistic drug–drug relationships have repeatedly been demonstrated using both simple correlation analysis and more sophisticated data analytical methods (Paull *et al*, 1989; Weinstein *et al*, 1992, 1997; Boyd and Paull, 1995). We have previously shown that, by applying similar techniques, a smaller panel of 10 cell lines representing different drug-resistant phenotypes could accomplish accurate classifications of mechanisms of action for common anticancer drugs (Dhar *et al*, 1996).

The development of novel molecular technologies such as cDNA microarrays has made it possible to identify genes involved in chemosensitivity. Integration of gene expression and drug activity data sets for cancer cells can identify relationships between individual genes and sensitivity or resistance to specific drugs. Investigators at NCI analysed the gene expression profiles of the NCI human tumour cell line panel and correlated the gene expression to growth-inhibitory activity of anticancer compounds (Scherf *et al*, 2000).

Several genes were identified which could be considered as candidate targets or biomarkers for chemosensitivity. The approach was considered feasible and useful for exploring the mechanisms of action, and was supported by investigators applying a similar methodology on a 39-cell line panel (Dan *et al*, 2002). The cell lines used in these studies consisted of parental cells of different cancer types and did not include any selected resistant phenotypes. With the aim of identifying chemosensitivity genes, the inclusion of resistant cell lines may be advantageous by increasing the range of expression in the measured microarray data, specifically for the genes involved in development of resistance. Therefore, in the present study, a cell line panel representing different drug resistance phenotypes, rather than histological origin, was characterised with respect to gene expression and anticancer drug response, and the relationships between the resulting drug and gene expression profiles were subsequently explored. By association analyses using pathway mining software, molecular pathways putatively involved in drug resistance and sensitivity were identified.

## MATERIALS AND METHODS

### Cell culture

The human cancer cell line panel has been described previously (Dhar *et al*, 1996). The panel consists of the parental cell lines RPMI 8226 (myeloma), CCRF-CEM (leukaemia), U937-GTB (lymphoma) and NCI-H69 (small-cell lung cancer); the drug-resistant sublines 8226/Dox40 8226/LR5, CEM/VM-1, U937/vcr, H69AR and the primary resistant ACHN (renal adenocarcinoma). 8226/Dox40 was exposed to 0.24 *μ*g ml^−1^ of doxorubicin once a month, and overexpresses Pgp/MDRl/ABCBl (Dalton *et al*, 1986). 8226/LR5 was exposed to 1.53 *μ*g ml^−1^ of melphalan at each change of medium, and the resistance is proposed to be associated with increased levels of glutathione as well as genes involved in cell cycle and DNA repair (Bellamy *et al*, 1991; Mulcahy *et al*, 1994; Hazlehurst *et al*, 2003). U937 vcr was continuously cultured in the presence of 10 ng ml^−1^ vincristine, and the resistance is proposed to be rubulin associated (Botling *et al*, 1994). H69AR was alternately fed with drug-free medium and medium containing 0.46 *μ*g ml^−1^ of doxorubicin, and overexpresses MRP1/ABCC1 (Mirski *et al*, 1987; Cole *et al*, 1992; Slovak *et al*, 1993). CEM/VM-1 was cultured in drug-free medium and could be grown for 3–4 months without loss of resistance against teniposide, which is proposed to be topoisomerase II associated (Danks *et al*, 1987, 1988; Mao *et al*, 1999). The primary drug resistance of ACHN is probably multifactorial (Nygren and Larsson, 1991). All cells were grown in culture medium RPMI-1640 supplemented with 10% heat-inactivated foetal calf serum, 2 mM glutamine, 100 *μ*g ml^−1^ streptomycin and 100 U ml^−1^ penicillin (all from Sigma Aldrich Co, St Louis, MO, USA) at 37°C in humidified air containing 5% CO_2_. The resistant cell lines were tested regularly for maintained resistance to the selected drugs. Growth and morphology of all cell lines were monitored on a weekly basis.

### Measurement of drug activity

A total of 66 anticancer drugs (Table 1), obtained from commercial sources or from NCI, were dissolved according to the manufacturer's instructions and tested in five concentrations, obtained by 10-fold serial dilution. The investigational alkylating agents Jl and P2 were kind gifts from Oncopeptides AB (Stockholm, Sweden). The Fluorometric Microculture Cytotoxicity Assay (FMCA), described in detail previously (Larsson *et al*, 1990), is based on measurement of fluorescence generated from hydrolysis of fluoroscein diacetate (FDA) to fluorescein by cells with intact plasma membranes. Briefly, cells were seeded into microtitre plates (Nunc, Roskilde, Denmark) prepared with drugs and incubated at 37°C and 5% CO_2_. for 72 h. Then the plates were washed, FDA added, and, after 40 min of incubation, the fluorescence was measured in a Fluoroscan II (Labsystems Oy, Helsinki, Finland). The fluorescence is proportional to the number of living cells and data are presented as survival index, defined as the fluorescence of experimental wells in percent of control wells with blank values subtracted. The IC_50_ value for each drug in each cell line was obtained from concentration–response curves constructed in Excel (Microsoft) and GraphPadPrism (GraphPad Software Inc., CA, USA).

### RNA extraction and reference composition

Total RNA was extracted from each cell line starting from 10 cells, using Trizol reagent (Invitrogen, Carlsbad, CA, USA) according to the manufacturer's protocol. The purity of the RNA was ensured by measuring the optical density at 260 and 280 nm. The integrity of the RNA was controlled by capillary electrophoresis using a Bioanalyzer 2100 (Agilent Technologies, Palo Alto, CA, USA). Only pure RNA (OD 260/280 >1.8) without any sign of degradation was used in the subsequent experiments. The common reference RNA used in the array experiments was composed of equal aliquots from the cell lines HELA, ACHN, U937-GTB, HTERT-RPE and H69AR.

### Array fabrication

In all, 7458 cDNA clones, included in the Human Sequence Verified Set, were obtained from Research Genetics (Huntsville, AL, USA). A complete list of genes printed on the arrays is available at: http://www.genpat.uu.se/Forskargrupper/w cn/UU/InstrAndProd_section.htm#prod. Plasmids containing clones were grown in *Escherichia coli* overnight in 96-well microtitre plates. Plasmid DNA was isolated using the Millipore Plasmid Miniprep_96_ Kit (Millipore, Bedford, MA, USA) and clone inserts were amplified using vector-specific primers (Universal Forward 5′-CTGCAAGGCGATTAAGTTGGGTAAC-3′ and Universal Reverse 5′-GTGAGCGGATAACAATTTCACACAGGAAACAGC-3′). The PCR products were purified with the Millipore Multiscreen PCR 96-well plate filtration system (Millipore) and dissolved in 45 *μ*l MilliQ-water. The PCR products were dried and re-suspended in MilliQ-water, containing 30% DMSO, to a final concentration of 0.1 mg ml^−1^. The PCR products were printed with a Cartesian Prosys 5510A (Cartesian Technologies Inc., Irvine, CA, USA) in duplicates with eight 3B Stealthpins (TeleChem International Inc., Sunnyvale, CA, USA) on GAPSII slides (Corning Life Sciences, Acton, MA, USA). The printing temperature was 25°C and the relative humidity 65%. The spotted PCR products were crosslinked to the slides at 450 mJ using a Stratalinker UV 1800 (Stratagene, La Jolla, CA, USA).

### Probe preparation, hybridisation, development and image acquisition

Labelling and detection of cDNA were carried out using the TSA Labelling and Detection Kit (NEN Life Science Products, Boston, MA, USA). The TSA probe labelling, array hybridisation and development were performed as described previously (Karsten *et al*, 2002). The microarrays were scanned in a GenePix 4000B scanner (Axon Instruments, Union City, CA, USA) at wavelengths 635 and 532 nm for Cy5 and Cy3 dyes, respectively, using 10-*μ*m resolution.

### Image processing and normalisation and filtering

The images were analysed and raw data were extracted, using GenePix Pro software version 5.0. (Axon Instruments). Raw data were normalised using the SMA package (Statistics for Microarray Analysis: http://www.stat.berkeley.edu/users/terry/zarray/Software/smacode.html). The algorithm used was LOWESS print tip normalisation (Yang *et al*, 2002). Each cell line was analysed on two separate arrays with the dyes reversed, providing a total of four (genes printed in duplicates on each array) measurements per gene and cell line. Genes with missing values for more than half of the cell lines were removed from the data set. This filter reduced the number of genes from 7458 to 3903. For genes passing this filtering criteria, an average expression level for each gene and sample was calculated and used in further analysis.

### Data analysis

The drug- and gene-expression databases were integrated and a correlation analysis performed in a custom-made program with similar functions as COMPARE (http://www.nci-sw.com/compare.html). Pearson's correlation coefficients for all drug–drug (log 10 IC_50_), gene–gene (log 2) and drug–gene correlations (log 10, log 2) were automatically calculated and stored in this database. Differential drug activity and differential gene expression were displayed in delta graphs. The cell line panel mean log 10 IC_50_ or log 2 gene-expression values were determined and subtracted from the log 10 or log 2 values for each cell line to yield the variable defined as delta. Unsupervised hierarchical cluster analysis for cells–genes, cells–drugs and genes–drugs was performed with the CIMminer software (http://discover.nci.nih.Kov/nature2000/tools/cimmaker.isp) using average linkage clustering with Pearson's correlation coefficient as the measure of similarity. A correlation coefficient above 0.7 or below -0.70 was chosen to extract the genes specifically associated with drug sensitivity and resistance, respectively. This level of Pearson's correlation coefficient corresponds to a significance level of *P*<0.05 for a two-tailed test for 10 observation pairs of the null hypothesis that the correlation is zero.

### Identification of molecular pathways

The genes connected to general chemosensitivity and resistance were analysed using PathwayAssist software to identify signalling pathways (Pathway assist v3.0. (www.ariadnegenomics.com). PathwayAssist is a software for visualisation and exploration of biological pathways, gene regulation networks and protein–protein interactions. PathwayAssist is supplied with ResNet molecular interaction and pathway database, which contains more than 500 000 functional links for more than 50 000 proteins, extracted from more than 5 000 000 Medline abstracts and full-length articles (ResNet update Q4 2004).

## RESULTS

### Analysis of the gene expression and drug activity data sets

**Figure 1:**
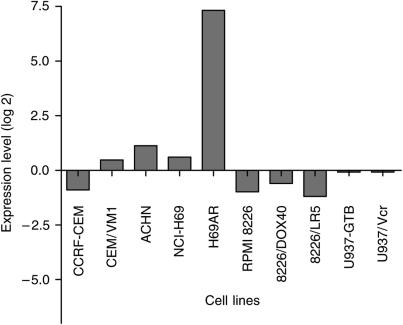
Differential expression of MRP1/ABCC1 in the cell line panel.

**Figure 2:**
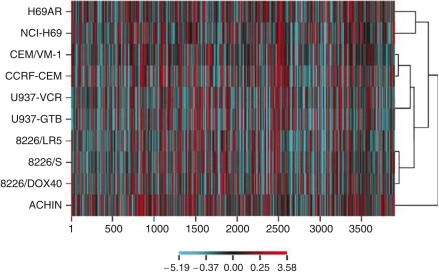
Two-dimensional hierarchical clustering analysis based on similarities in gene expression in the cell lines.

**Figure 3:**
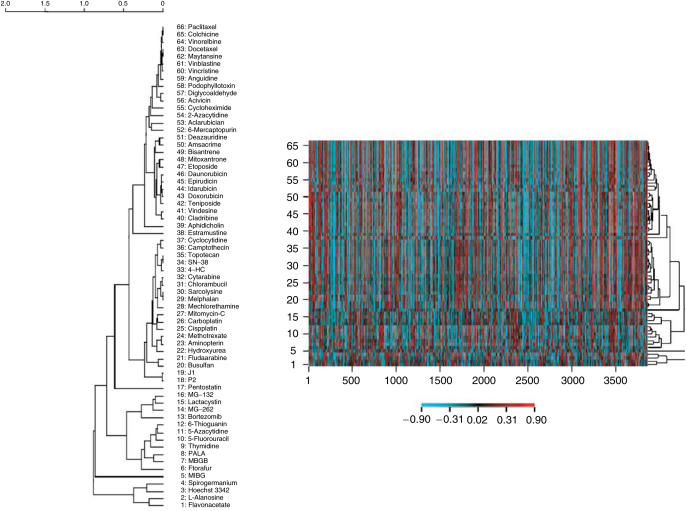
Two-dimensional hierarchical clustering analysis based on drug–gene correlations (Pearsons correlation coefficients) for drug response data (log 10 IC_50_) of 66 anticancer drugs and 3903 genes (log 2) in the 10 cell lines.

**Figure 4:**
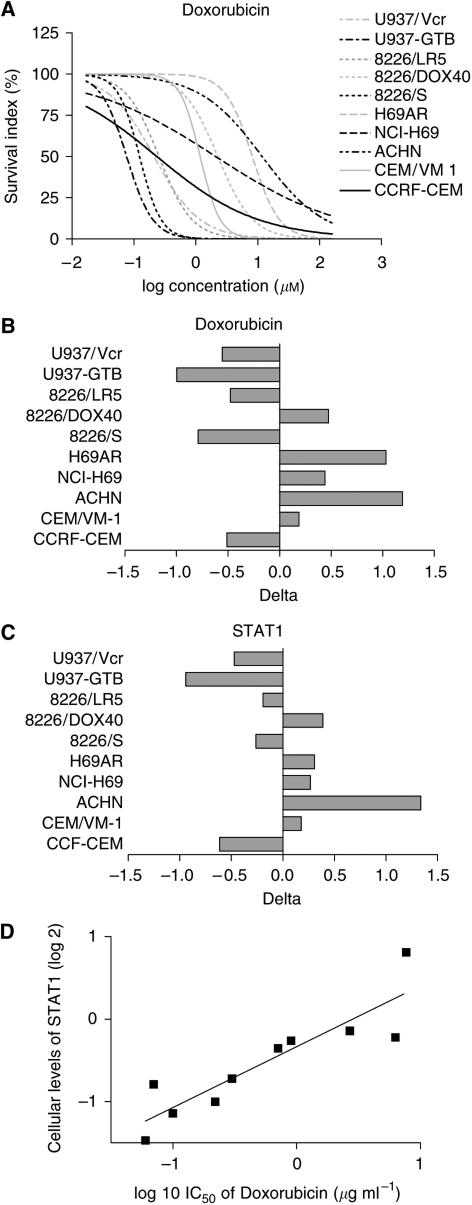
An example of the drug–gene correlations obtained. Concentration–response curves for doxorubicin in the cell line panel (**A**). Correlation between log 2 expression of STAT1 with log IC_50_ of doxorubicin (**B**). Mean graphs of doxorubicin (**C**) and STAT1 (**D**).

A cDNA microarray analysis was performed to investigate the expression profiles of the 7458 genes in each of the 10 cell lines. Of these genes, 3903 fulfilled the preset quality criteria for subsequent analyses. An example is shown in Figure 1, in which the accurate detection of the ABCC1 transporter in the cell lines is shown. In general, cDNA microarray expression data need to be validated to ensure that the correct gene expression has been measured. In this case, oligonucleotide arrays with a shorter more specific probe has been used to validate MRPl/ABCCl expression (correlation coefficient *r*>0.9, data not shown). H69AR showed an increased expression of MRPl/ABCCl compared to all other cell lines, which is consistent with previous results, further supporting the validity of the array measurements (Cole *et al*, 1992). A hierarchical clustering method was then applied to the gene expression in the cell lines (Figure 2). The parental cell lines clustered with their resistant sublines, indicating that no gross alteration in the gene expression profile resulted from the selection of the drug-resistant sublines. Next, correlations were established between the log 2 expression values of each of these 3903 genes and the log 10 IC_50_ values obtained for each of the 66 drugs included in the study. Hierarchical clustering of drugs based on these drug–gene correlations resulted in clusters consisting of drugs with similar modes of action (Figure 3). All proteasome inhibitors and topoisomerase I (Topi) inhibitors and most of the antitubulins, topoisomerase II (Top2) inhibitors and alkylating agents formed distinct clusters. Notable exceptions were the tubulin active agents vindesine and estramustine, which did not cluster within their assigned mechanistic group. The antimetabolites clustered more heterogeneously, but closely related drugs with respect to mechanism of action, such as the dihydrofolate reductase inhibitors methotrexate and aminopterin, clustered together. Also, Jl and P2, oligopeptide derivatives of melphalan and sarcolysine, respectively, did not cluster with their parent compounds. Clustering of drugs based on drug activity alone yielded similar results as the clustering based on the drug–gene correlations (data not shown). An example of a typical drug–gene relationship is shown in Figure 4 Concentration–response curves for doxorubicin in the cell line panel and delta graphs for differential drug activity and STAT1 gene expression are depicted in panels A–C, respectively. The activity (log 10 IC_50_) of doxorubicin and the expression of STAT1 (log 2) in the cell lines were highly correlated (panel D, *R*=0.89). Table 2 displays the 40 genes with the highest positive and negative correlations to doxorubicin.

### Identification of signalling pathways associated to drug sensitivity and resistance

**Figure 5:**
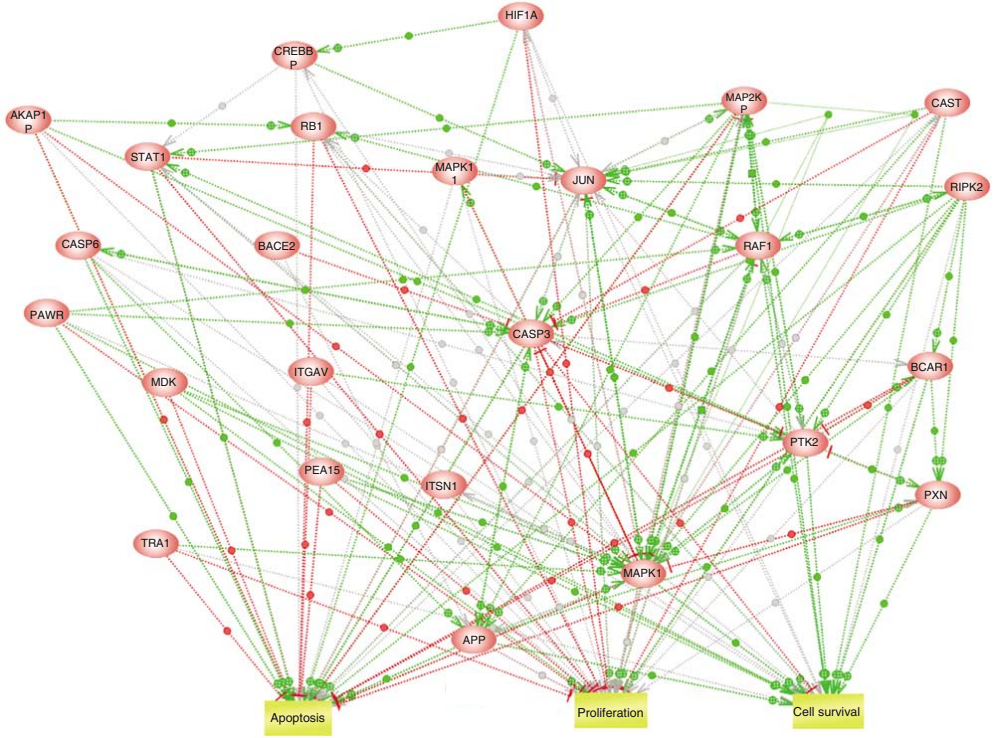
Analysis of molecular interactions using PathwayAssist. A simplified network for genes associated with resistance is shown. Green lines indicate positive effects, red lines indicate negative effects and grey lines interactions with unknown effect. Complete interactive graphical versions of the networks associated with sensitivity and resistance are accessible in supplementary information online and the connections are clickable (dots on lines) to access hyperlinks to the Medline references on which the networks are based. Clicking on the nodes provides hyperlinks to several gene and protein databases including HUGO, Locus Link and Swiss-Prot for the particular protein.

Genes where *R*>0.70. and <−0.70 were extracted for each of the 66 drugs (supplementary information online). A correlation coefficient of >0.70 or <−0.70 in at least 20 of the 66 drugs was set as the criterion for the selection of genes associated to chemosensitivity. This selection identified 122 and 74 genes correlated to general resistance and sensitivity, respectively. Next, Pathway Assist was used in two steps to explore the interactions between the genes on the two lists. In the first step, the ResNet database was searched to establish direct interactions between the genes, and, in the second step, the genes were searched for linkage to cellular processes involving cellular proliferation, cell survival, cell death or apoptosis. The genes selected in this way to form these molecular networks are listed in Tables 3 and 4. The two networks are accessible in supplementary information online as html web files, and the connections are clickable (dots on lines) to access hyperlinks to the Medline references on which the networks are based. Clicking on the nodes provides hyperlinks to several gene and protein databases, including HUGO, OMIM, Locus Link and Swiss-Prot for the particular protein. A simplified version of the network for genes associated with drug resistance is shown in Figure 5. Regarding genes associated to sensitivity, there was a considerable number that could be linked to cell cycle and proliferation regulation rather than apoptosis, including genes such as CDC25A, CCNC, CCND3 and STAT5A (Figure 5A). Notably, for the resistance associated genes, both known proapoptotic and antiapoptotic pathways were detected in the resulting network (Figure 5B). In this molecular network, caspase 3 and 6 and Jun were identified together with survival genes such as RB1, calpastatin, PTK2 and MAPK.1. These gene/pathway maps may provide novel potential molecular targets for therapy. In addition to the genes selected by Pathway Assist, several other potentially relevant genes fulfilled the general resistance and sensitivity criteria including ABC transporters, drug-inactivating enzymes and protein kinases (for complete general sensitivity and resistance lists, see the supplementary information online).

## DISCUSSION

Gene–drug relationships in large panels of cancer cell lines with different histological origins have been studied previously (Scherf *et al*, 2000; Dan *et al*, 2002). In the present study, we studied the gene expression and drug activity in a panel of 10 cell lines representing different mechanisms of anticancer drug resistance. Previous studies have shown that drug activity patterns in this panel can be used to classify anticancer drugs according to mechanism of action (Dhar *et al*, 1996). Here we showed that this classification also corresponded to identifiable patterns of gene expression and that the genes which correlated to drug sensitivity and resistance seem to be biologically relevant. The gene-expression profiles of the cell lines were similar for cells with the same histological origin and the hierarchical clustering performed based on drug–gene correlations for the drugs in the cell lines yielded clusters of drugs based on their main mechanism of action, with some exceptions. There are several possible reasons for incorrect clustering, and these include experimental variability and incorrect or incomplete assignment of the mechanism of action. Concerning the antimetabolites, the clustering was not clearly linked to known structural or mechanistic features. Given the very diverse mechanistic properties of these drugs, this was not an unexpected finding. Other drugs that deviated from the expected clustering were vindesine, estramustine, J1 and P2. As an example, Jl and P2, oligopeptide derivatives of melphalan and sarcolysine, respectively, clustered together, but differently from their parent compounds. Jl is currently undergoing clinical development, and recent studies have indicated other mechanisms of cell death additional to the death caused by DNA alkylation (Gullbo *et al*, 2003). The overall results indicate that the panel of 10 tumour cell lines was able to reasonably well classify drugs with respect to the mechanism of action.

The mechanistic pathways identified with PathwayAssist associated to general drug resistance paradoxically included a substantial number of both proapoptotic and antiapoptotic genes. The proapoptotic genes caspase 3 and 6 (CASP3, 6) and Jun were identified together with survival genes such as retinoblastoma 1 (RBI), calpastatin (CAST), focal adhesion kinase/protein tyrosine kinase 2 (FAK/PTK2) and mitogen-activated protein kinase 1 (MAPK1). A parallel upregulation of pro- and antiapoptotic genes in malignant tumours has been observed in several microarray studies comparing tumour cells and normal tissue (Rhodes *et al*, 2004; www.oncomine.com), indicating that the balance between upregulated pro- and antiapoptotic genes may be critical for tumour cell survival. To affect this balance by small molecules may thus be a potential therapeutic strategy. The expression of signal transducer and activator of transcription 1 (STATl) was also observed to be highly correlated to resistance, particularly for the Top2 inhibitors (data not shown). Although activation of STATl in some cell systems has been shown to be proapoptotic (Calo *et al*, 2003), a recent observation has indicated a role for STATl in mediating radiation resistance (Khodarev *et al*, 2004). Recently, a correlation between STATl expression and cisplatin resistance in cell lines derived from patients with ovarian carcinoma was also reported (Roberts *et al*, 2005), and inhibitors of the STATl pathway have been shown to induce apoptosis in leukaemic cells from patients (Martinez-Lostao *et al*, 2005). The pathway analysis showed that STATl is positively influenced by MAPK1 and FAK, two of the most highly connected resistance-associated genes, both of which have been reported to inhibit apoptosis (Shimada *et al*, 2002; Kurenova *et al*, 2004). Notably, STATl expression has been reported higher in tumour compared with corresponding normal tissue for a wide range of tumour types (www.oncomine.com). The STATl pathway may thus provide a potentially interesting drug target for reversal of drug resistance.

The genes correlated to drug sensitivity had diverse functions, but a considerable number were found to be related to cell cycle and proliferation rather than to apoptosis, for example, cell division cycle 25A (CDC25A) and signal transducer of activator of transcription 5A (STAT5A). This is in accordance with the general notion of a correlation between high proliferation and increased anticancer drug sensitivity (Valeriote and van Putten, 1975; Kaaijk *et al*, 2003).

Some limitations of the study should be discussed. First, cell lines removed from their *in vivo* environment and selected for growth in culture differ from tumour cells in patients. Therefore, the relevance of the genes and mechanistic pathways needs to be studied in additional settings, such as primary cultures of tumour cells from patients. Second, the drug activity database was generated using a single assay end point, that is, short-term growth inhibition and cytotoxicity. Drugs may induce concentration-dependent effects on different targets leading to different modes of cell death, including apoptosis, necrosis and cell senescence (Blagosklonny, 2004). Multiparameter assays using high-content screening may provide a substantial increase in the information on drug activity and mode of cell death (Lövborg *et al* 2004), and such studies are underway. Third, only a part of all human genes were represented on the arrays used. Also, the gene–drug relationships described represent only a small fraction of relationships thought to be relevant to chemotherapy, and many are probably hidden by the arbitrary and rough cutoff criteria that need to be applied for selection of the information, among all the available microarray data, considered to be relevant. Fourth, with respect to the data exploration, the quality of the data obtained is dependent on the information and algorithms in the software used, meaning that these data should only be used for generation of hypotheses that need further and direct confirmation. Finally, although this exploitation of array data using powerful software can provide a basis for identification of new drug targets, it should be emphasised that the relationships observed are correlative, not causal, and that these correlations must be further experimentally tested and validated.

In comparison to previous similar studies (Scherf *et al*, 2000; Dan *et al*, 2002), we identified other chemosensitivity genes. This might be explained by several limiting factors as discussed above, and include differences in cell types, arrays, drugs and assays used. Even when different arrays were used to study the same samples, the gene–drug relationships were shown to differ (Scherf *et al*, 2000; Staunton *et al*, 2001). Furthermore, in the present study, the correlation coefficients for the drug–gene correlations were in general higher than in the previous studies. This might partly be explained by the inclusion of cell lines selected for drug resistance. The selection of drug resistance may impose a larger range of gene expression across samples, leading to higher drug–gene correlation coefficients. This would also potentially increase the possibility of identifying genes specifically associated with drug resistance. An advantage of using parental and drug-resistant cell lines is that the selecting agent and genes specifically involved in resistance to that particular drug could be isolated. However, it should be noted that the ability to cluster drugs based on drug–gene correlations is not limited to drug classes used for drug resistance selection. Indeed, in the present paper, both the proteasome inhibitors and Top I inhibitors could be distinguished as distinct clusters, although no resistant cell lines representative of these drug classes were included in the panel. Extending the panel with sublines resistant to mechanistically different drugs may nevertheless improve the possibility of identifying genes involved in drug resistance by further increasing the range of relevant gene expression. We are currently introducing cell lines resistant to novel target-specific drugs such as proteasome and tyrosine kinase inhibitors.

In the present study, we employed a molecular pathway analysis tool, Pathway Assist (www.ariadnegenomics.com), for the analysis of obtained drug–gene correlations. This procedure allowed quick and efficient generation of biologically meaningful and literature-validated relationships between the genes retrieved. PathwayAssist contains the ResNet database in which more than 500 000 events are recorded and any established pathway can be updated online by automated mining of Medline using a built-in Natural Language Processing algorithm (www.ariadnegenomics.com). Performing such pathway analysis manually would have been extremely time consuming and laborious. Automated data-mining tools for pathway analysis will therefore be increasingly important in light of the exploding information content on molecular pathway networks.

In conclusion, integration of gene expression and drug activity data sets for tumour cell line panels provide relationships between individual gene and drug activity profiles that makes it possible to identify drug mechanisms of action that can be traced down to gene level. By also applying powerful software for recognition of cell signalling pathways, the current approach might accelerate the drug discovery and evaluation process and provide novel markers and drug targets for the chemotherapy of cancer. The current approach is suitable for characterisation of new drugs both with respect to the mechanism of action and identification of genes involved in drug sensitivity and resistance.

## Figures and Tables

**Table 1:** Anticancer drugs used in the study

*Antimetabolites*
Acivicin, Aminopterin, Aphidicolin, 5-Azacytidine, L-Alanosine , Cladribine, Cyclocytidine, Cytarabine, 3-Deazauridine, 2-Azacytidine, Diglycoaldehyde, Fludarabine, 5-Fluorouracil, Ftorafur, Hydroxyurea, 6-Mercaptopurine Methotrexate, PALA, Pentostatin, 6-Thioguanine, Thymidine

*Alkylating agents*
Busulfan, Carboplatin, Chlorambucil, Cisplatin, 4-HC, J1, Mechlorethamine, Melphalan, Mitomycin C, P2, Sarcolysine

*Topoisomerase I-inhibitors*
Camptothecin, SN-38, Topotecan

*Topoisomerase II-inhibitors*
Amsacrine, Bisantrene, Daunorubicin, Doxorubicin, Epirubicin, Etoposide, Idarubicin, Mitoxantrone Teniposide

*Proteasome inhibitors*
Bortezomib, Lactacystin, MG-132, MG-262

*Tubulin active agents*
Colchicine, Docetaxel, Maytansine, Paclitaxel, Podophyllotoxin, Vinblastine, Vincristine, Vindesine, Vinorelbine, Estramustine

*Others*
Aclarubicin, Anguidine, Cycloheximide, Flavoneacetate, Hoechst 33342, MBGB, MIBG, Spirogermanium

PALA=*N*-phosphonacetyl-L-aspartate; 4-HC=4-hydroperoxy-cyclophosphamide; MGBG=methylglyoxal-bis(guanylhydrazone); MIBG=meta-iodobenzylguanidine; SN-38=active metabolite of camptothecin; J1 and P2, oligopeptide derivatives of melphalan and sarcolysine, respectively.

**Table 2:** The genes with highest positive and negative correlations to doxorubicin

**Symbol**	**Acc ID**	**Name**	***R***
CTTN	M98343	Cortactin	0.96
SLC39A1	BC047288	Solute carrier family 39 (zinc transporter), member 1	0.93
TTF2	NM_003594	Transcription termination factor, RNA polymerase II	0.93
TPI1	BM913099	Triosephosphate isomerase 1	0.93
DDAH1	NM_012137	Dimethylarginine dimethylaminohydrolase 1	0.92
EXTL1	NM_004455	Exostoses (multiple)-like 1	0.92
GNA11	BC063426	Guanine nucleotide binding protein (G protein), alpha 11	0.92
MAPK11	BC027933	Mitogen-activated protein kinase 11	0.92
SURB7	NM_004264	SRB7 suppressor of RNA polymerase B homolog (yeast)	0.91
HLA-DOA	NM_002119	Major histocompatibility complex, class II, DO alpha	0.90
STAT1	NM_007315	Signal transducer and activator of transcription 1, 91 kDa	0.89
AGRN	AB191264	Agrin	0.89
PTTG1IP	AK095586	Pituitary tumour-transforming 1 interacting protein	0.89
FXYD1	AK124802	FXYD domain containing ion transport regulator 1	0.88
DRPLA	BC051795	Dentatorubral-pallidoluysian atrophy	0.88
TULP3	NM_003324	Tubby like protein 3	0.88
BCAR1	AK124526	Breast cancer anti-oestrogen resistance 1	0.88
SPAG9	AF327452	Sperm associated antigen 9	0.87
ABCB6	BC043423	ATP-binding cassette, subfamily B (MDR/TAP), member 6	0.87
CAST	NM_173060	Calpastatin	0.87
BNIP2	AK125533	BCL2/adenovirus E1B 19 kDa interacting protein 2	−0.97
GCAT	AK123190	Glycine C-acetyltransferase	−0.92
TIMM10	BQ011318	Translocase of inner mitochondrial membrane 10 homolog (yeast)	−0.91
RNUT1	BG421329	RNA, U transporter 1	−0.90
ASCC3	AL834463	DJ467N11.1 protein	−0.90
GAS7	NM_201433	Growth arrest-specific 7	−0.88
CORO1A	AK123401	Coronin, actin-binding protein, 1A	−0.88
LCP2	NM_005565	Lymphocyte cytosolic protein 2	−0.88
WAS	BM455138	Wiskott–Aldrich syndrome	−0.88
EBP	BE253850	Emopamil-binding protein	−0.88
PSD4	BC073151	Pleckstrin and Sec7 domain containing 4	−0.87
KIAA1545	AB046765	KIAA1545 protein	−0.87
IDH3A	AK123316	Isocitrate dehydrogenase 3 (NAD+) alpha	−0.87
NIPBL	AJ627032	Nipped-B homolog (*Drosophila*)	−0.86
POLQ	NM_006596	Polymerase (DNA directed), theta	−0.86
C15orf22	AK075529	Chromosome 15 open reading frame 22	−0.85
RAC2	AK096924	Ras-related C3 botulinum toxin substrate 2	−0.85
PHF2	NM_005392	PHD finger protein 2	−0.85
TPBG	NM_006670	Trophoblast glycoprotein	−0.85
ARHGDIB	AK125625	Rho GDP dissociation inhibitor (GDI) beta	−0.84

**Table 3:** Genes associated with drug resistance selected by PathwayAssist

**Symbol**	**Name**
AGRN	Agrin
APP	Amyloid beta (A4) precursor protein (protease nexin-II, Alzheimer's disease)
BACE2	Beta-site APP-cleaving enzyme 2
BAG3	Bcl2-associated athanogene 3
BASP1	Brain abundant, membrane-attached signal protein 1
BCAR1	Breast cancer antioestrogen resistance 1
BMPR1A	Bone morphogenetic protein receptor, type IA
CASP3	Caspase 3, apoptosis-related cysteine protease
CASP6	Caspase 6, apoptosis-related cysteine protease
CAST	Calpastatin
CD9	CD9 antigen (p24)
CKAP4	Cytoskeleton-associated protein 4
CREBBP	CREB-binding protein
CSPG2	Chondroitin sulphate proteoglycan 2 (versican)
CTTN	Cortactin
DAG1	Dystroglycan 1 (dystrophin-associated glycoprotein 1)
DDAH1	Dimethylarginine dimethylaminohydrolase 1
DRPLA	Dentatorubral-pallidoluysian atrophy (atrophin-1)
EPHA2	EphA2
FXYD1	FXYD domain containing ion transport regulator 1 (phospholemman)
HIF1A	Hypoxia-inducible factor 1, alpha subunit (basic helix–loop–helix transcription factor)
IGSF4	Immunoglobulin superfamily, member 4
ITGAV	Integrin, alpha V (vitronectin receptor, alpha polypeptide, antigen CD51)
ITPR3	Inositol 1,4,5-triphosphate receptor, type 3
ITSN1	Intersectin 1 (SH3 domain protein)
JUN	Jun oncogene
LTBP1	Latent transforming growth factor beta binding protein 1
MAFG	V-maf musculoaponeurotic fibrosarcoma oncogene homologue G (avian)
MAP4K3	Mitogen-activated protein kinase kinase kinase kinase 3
MAPK1	Mitogen activated protein kinase 1
MAPK11	Mitogen-activated protein kinase 11
MDK	Midkine (neurite growth-promoting factor 2)
NBL1	Neuroblastoma, suppression of tumorigenicity 1
NEFL	Neurofilament, light polypeptide 68 kDa
PAWR	PRKC, apoptosis, WT1, regulator
PEA15	Phosphoprotein enriched in astrocytes 15
PLXNB1	Plexin B1
POR	P450 (cytochrome) oxidoreductase
PTK2	PTK2 protein tyrosine kinase 2
PTPN13	Protein tyrosine phosphatase, nonreceptor type 13
PXN	Paxillin
RB1	Retinoblastoma 1
RDX	Radixin
RIPK2	Receptor-interacting serine–threonine kinase 2
S100A10	S100 calcium-binding protein A10 (annexin II ligand, calpactin I, light polypeptide (p11))
SERPINH1	Serine (or cysteine) proteinase inhibitor, clade H (heat shock protein 47), member 1 (collagen-binding protein 1)
STAT1	Signal transducer and activator of transcription 1
TPI1	Triosephosphate isomerase 1
TRA1	Tumour rejection antigen (gp96) 1
TUBB	Tubulin, beta polypeptide
TULP3	Tubby like protein 3

The listed genes were selected by the PathwayAssist software as described in the Results section. An interactive graphical version is available in the supplementary information online.

**Table 4:** Genes associated with drug sensitivity selected by PathwayAssist

**Symbol**	**Name**
ARHGDIB	Rho GDP dissociation inhibitor (GDI) beta
BCCIP	BRCA2 and CDKN1A interacting protein
CCNC	Cyclin C
CCND3	Cyclin D3
CD37	CD37 antigen
CD4	CD4 antigen
CDX2	Caudal type homeo box transcription factor 2
CKLF	Chemokine-like factor
CORO1A	Coronin, actin-binding protein, 1A
DOCK2	Dedicator of cytokinesis 2
GAS7	Growth arrest-specific 7
GNA15	Guanine nucleotide-binding protein (G protein), alpha 15 (Gq class)
HDAC1	Histone deacetylase 1
IDH3A	Isocitrate dehydrogenase 3 (NAD+) alpha
IL2RG	Interleukin 2 receptor, gamma (severe combined immunodeficiency)
IMPDH2	IMP (inosine monophosphate) dehydrogenase 2
INPP5D	Inositol polyphosphate-5-phosphatase D
LCP1	Lymphocyte cytosolic protein 1 (L-plastin)
LCP2	Lymphocyte cytosolic protein 2 (SH2 domain containing leukocyte protein of 76 kDa)
MAP4K1	Mitogen-activated protein kinase kinase kinase kinase 1
MCM5	MCM5 minichromosome maintenance deficient 5, cell division cycle 46 (*S. cerevisiae*)
MYB	V-myb myeloblastosis viral oncogene homologue (avian)
MYCBP2	MYC binding protein 2
NCF4	Neutrophil cytosolic factor 4, 40 kDa
NUDC	Nuclear distribution gene C homolog (*A. nidulans*)
PENK	Proenkephalin
PPIH	Peptidyl prolyl isomerase H (cyclophilin H)
RAC2	Ras-related C3 botulinum toxin substrate 2 (rho family, small GTP-binding protein Rac2)
SLC25A5	Solute carrier family 25 (mitochondrial carrier; adenine nucleotide translocator), member 5
SLC7A5	Solute carrier family 7 (cationic amino-acid transporter, y+ system), member 5
STAT5A	Signal transducer and activator of transcription 5A
TFR2	Transferrin receptor 2
TPBG	Trophoblast glycoprotein
WAS	Wiskott–Aldrich syndrome (eczema-thrombocytopenia)

The listed genes were selected by the PathwayAssist software as described in the Results section. An interactive graphical version is available in the supplementary information online.
